# mGluR1-Mediated Excitation of Cerebellar GABAergic Interneurons Requires Both G Protein-Dependent and Src–ERK1/2-Dependent Signaling Pathways

**DOI:** 10.1371/journal.pone.0106316

**Published:** 2014-09-02

**Authors:** Hideo Kubota, Soichi Nagao, Kunihiko Obata, Moritoshi Hirono

**Affiliations:** 1 Materials Management, Medical Hospital, Tokyo Medical and Dental University (TMDU), Bunkyo, Tokyo, Japan; 2 Laboratory for Motor Learning Control, RIKEN Brain Science Institute, Wako, Saitama, Japan; 3 Obata Research Unit, RIKEN Brain Science Institute, Wako, Saitama, Japan; Indiana University School of Medicine, United States of America

## Abstract

Stimulation of type I metabotropic glutamate receptors (mGluR1/5) in several neuronal types induces slow excitatory responses through activation of transient receptor potential canonical (TRPC) channels. GABAergic cerebellar molecular layer interneurons (MLIs) modulate firing patterns of Purkinje cells (PCs), which play a key role in cerebellar information processing. MLIs express mGluR1, and activation of mGluR1 induces an inward current, but its precise intracellular signaling pathways are unknown. We found that mGluR1 activation facilitated spontaneous firing of mouse cerebellar MLIs through an inward current mediated by TRPC1 channels. This mGluR1-mediated inward current depends on both G protein-dependent and -independent pathways. The nonselective protein tyrosine kinase inhibitors genistein and AG490 as well as the selective extracellular signal-regulated kinase 1/2 (ERK1/2) inhibitors PD98059 and SL327 suppressed the mGluR1-mediated current responses. Following G protein blockade, the residual mGluR1-mediated inward current was significantly reduced by the selective Src tyrosine kinase inhibitor PP2. In contrast to cerebellar PCs, GABA_B_ receptor activation in MLIs did not alter the mGluR1-mediated inward current, suggesting that there is no cross-talk between mGluR1 and GABA_B_ receptors in MLIs. Thus, activation of mGluR1 facilitates firing of MLIs through the TRPC1-mediated inward current, which depends on not only G protein-dependent but also Src–ERK1/2-dependent signaling pathways, and consequently depresses the excitability of cerebellar PCs.

## Introduction

Inhibitory interneurons play a crucial role in regulating the function of various neuronal networks in the central nervous system [1]–[3]. Therefore, it is important to clarify the precise mechanisms, by which neurotransmitters such as glutamate, GABA, and monoamines modulate the excitability of inhibitory interneurons. In the cerebellum, GABAergic synaptic inhibition onto Purkinje cells (PCs) modulates firing patterns of PCs and regulates cerebellar information processing [3]–[10]. It has been reported that activation of group I metabotropic glutamate receptors (mGluR1/5) preferentially modulates synaptic transmission at postsynaptic sites [11], [12]. By contrast, inhibitory synaptic transmission onto PCs is facilitated by presynaptic mGluR1 activation, which increases the spontaneous firing rate of the two types of inhibitory interneurons located in the molecular layer (MLIs), basket cells and stellate cells [13], [14]. However, the roles of presynaptic group I mGluRs are less clear.

Previous studies reported that G protein-independent pathways are involved in excitatory responses elicited by activation of group I mGluRs in hippocampal neurons [15]–[17]. In hippocampal CA3 pyramidal neurons, activation of the G protein-independent protein tyrosine kinase (PTK) Src family contributes to mGluR1-mediated excitatory responses [15]. In addition, in hippocampal oriens/alveus inhibitory interneurons, group I mGluR activation elicits the activation of the Src–extracellular signal-regulated kinase 1/2 (ERK1/2) cascade [17]. On the other hand, mGluR1 activation in cerebellar PCs evokes a similar excitatory inward current through a G protein-dependent and phospholipase C (PLC)-independent signaling pathway [18]–[20], and type 3 transient receptor potential canonical (TRPC3) channel activation is necessary for these current responses [21]–[25]. However, the underlying mechanism for mGluR1-mediated excitatory responses in MLIs remains unknown.

Here, we examined the molecular mechanisms underlying the mGluR1-mediated excitatory inward current in mouse cerebellar MLIs. We found that the group I mGluR-mediated inward current was markedly suppressed by blockade of mGluR1 or TRPC1. Moreover, the mGluR1-mediated inward current was partially inhibited by a selective inhibition of G proteins, Src, or ERK1/2. GABA_B_ receptor activation did not alter the mGluR1-mediated inward current, suggesting that there is no cross-talk between the signaling cascades following the activation of mGluR1 and GABA_B_ receptor signaling cascades. These results suggest that the mGluR1-mediated inward current in cerebellar MLIs is mediated by both G protein-dependent and G protein-independent Src–ERK1/2 signaling pathways.

## Materials and Methods

### Slice preparation

Cerebellar slices from C57BL/6 mice aged 18–25 days were prepared as previously described [20]. All experimental procedures were conducted in accordance with the National Institutes of Health Guide for the Care and Use of Laboratory Animals (NIH Publications No. 80-23; revised 1996). The RIKEN Animal Research Committee approved the procedures, and all efforts were made to minimize the number of animals used and their suffering. The C57BL/6 mice were deeply anesthetized with halothane, and 230-µm thick sagittal slices of cerebellar vermis were cut using a vibrating microtome (VT1000S, Leica, Nussloch, Germany) in an ice-cold extracellular solution containing (in mM) 252 sucrose, 3.35 KCl, 21 NaHCO_3_, 0.6 NaH_2_PO_4_, 9.9 glucose, 1 CaCl_2_, and 3 MgCl_2_ and gassed with a mixture of 95% O_2_ and 5% CO_2_ (pH 7.4). The slices were maintained at 30°C for 30 min in a holding chamber, where they were submerged in artificial cerebrospinal fluid (ACSF) containing (in mM) 138.6 NaCl, 3.35 KCl, 21 NaHCO_3_, 0.6 NaH_2_PO_4_, 9.9 glucose, 2 CaCl_2_, and 1 MgCl_2_, and aerated with 95% O_2_ and 5% CO_2_ to maintain the pH at 7.4. Thereafter, slices were maintained at room temperature.

### Electrophysiological recordings

Individual cerebellar slices were transferred to a recording chamber attached to the stage of a microscope (BX51WI, Olympus, Tokyo, Japan) and superfused with oxygenated ACSF. Basket/stellate cells (MLIs) were visually identified under Nomarski optics using a water immersion objective (60×, NA 0.90, Olympus). Extracellular spike activity in MLIs was observed by a loose cell-attached voltage-clamp at a holding potential of 0 mV. Glass electrodes used for cell-attached recordings had resistances of 3–4 MΩ when filled with ACSF. Whole-cell patch pipettes (2–3 MΩ) were filled with an intracellular solution containing (in mM) 120 KCH_3_SO_3_, 25 KCl, 0.1 CaCl_2_ 1.0 K–EGTA, 10.0 Na–HEPES, 3.0 Mg–ATP, and 0.4 Na–GTP (pH 7.4). In a subset of experiments, Na–GTP was substituted with the nonhydrolizable GTP analogue GDPβS (trilithium salt, 300 µM or 1 mM). MLIs were voltage-clamped at −70 to −65 mV. To specifically isolate the (*S*)-3,5-dihydroxyphenylglycine (DHPG)-induced slow inward currents from fast postsynaptic currents, the nonselective ionotropic glutamate receptor antagonist kynurenic acid (1 mM), the GABA_A_ receptor antagonist bicuculline (10 µM) (or 10 µM SR95531), and tetrodotoxin (TTX, 0.5 µM) were added to ACSF. In addition, the cannabinoid antagonist AM251 (2 µM) was added to ACSF for all the recordings. Membrane currents were recorded in whole-cell configuration using the MultiClamp 700B amplifier (Molecular Devices, Sunnyvale, CA) under the control of the pCLAMP 9.2 software (Molecular Devices). Recordings were digitized and stored on a computer disk for off-line analysis. All signals were filtered at 2 kHz and sampled at 5 kHz. Series resistance (8–14 MΩ) was monitored by the current response to a 2 mV hyperpolarizing voltage pulse (30–50 ms) delivered every 30 s, and recordings were discarded if the value changed by more than 20%. DHPG (300 µM) was applied every 40 s by pressure puff (8–10 psi, 150–250 ms duration) through microelectrodes placed in the vicinity of the MLI soma during recordings were obtained. All experiments were performed at room temperature (23–26°C).

### Drugs

DHPG, 7-(hydroxyimino)cyclopropa-[b]chromen-1a-carboxylate ethyl ester (CPCCOEt), (*S*)-(+)-α-amino-4-carboxy-2-methylbenzeneacetic acid (LY367385), 1-[2-(4-methoxyphenyl)-2-[3-(4-methoxyphenyl)propoxy]ethyl-1*H*-imidazole (SKF96365), (E)-2-cyano-3-(3,4-dihydrophenyl)-N-(phenylmethyl)-2-propenamide (AG490), (-)-bicuculline methochloride, 3-(4-chlorophenyl) 1-(1,1-dimethylethyl)-1*H* -pyrazolo[3,4-d]pyrimidin-4-amine (PP2), 1-phenyl-1H-pyrazolo[3,4-d]pyrimidin -4-amine (PP3), SR95531 (gabazine), U73122, U73343, and SL327 were obtained from Tocris Bioscience (Bristol, UK); 2-aminoethoxydiphenylborane (2-APB) was obtained from Abcam Biochemicals (Cambridge, UK); A polyclonal TRPC1 antibody and its control antigen, amino acid residues 557-571 of human TRPC1 were obtained from Alomone Labs (Cat#: ACC-010, Jerusalem, Israel). TTX was obtained from Wako (Osaka, Japan). All other chemicals were from Sigma (St. Louis, MO). Drugs were dissolved in dH_2_O or DMSO, according to the solubility advice of the manufacturer. The final concentration of DMSO did not exceed 0.1%.

### Data analysis and statistics

Spike firing and the DHPG-induced inward currents were analyzed using Mini Analysis Program 6.0 (Synaptosoft, Decatur, GA), pCLAMP 9.2 software, and Kyplot software 5.0 (KyensLab, Tokyo, Japan). All data are expressed as mean ± standard error of the mean (SEM). Unless otherwise stated, the level of significance was determined by paired Student's *t*-test between groups. For time course analyses of the effect of bath-applied drugs (except for baclofen), the amplitude of the DHPG-induced inward current is expressed as a percentage of the average control current during the 10 min before application. For time course analyses of the effect of drugs dialyzed into MLIs, the amplitude of the DHPG-induced inward current is expressed as a percentage of the current obtained 2 min after the initiation of the whole-cell configuration (patch membrane rupture).

## Results

**Figure 1:**
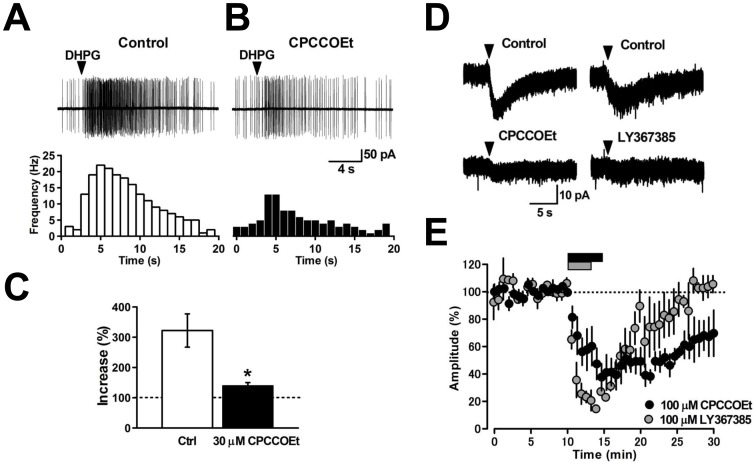
Group I mGluR agonist DHPG facilitates spontaneous firing and induces excitatory inward current in cerebellar MLIs. (A) Puff-application of DHPG (arrowhead) increased the firing rate of a MLI under control conditions (upper). Time course of the firing rate (lower). The average firing rate calculated for 1-s bins was plotted at each time point. (B) A non-competitive mGluR1 antagonist CPCCOEt (30 µM) blocked the DHPG-induced firing facilitation (upper). Time course of the firing rate with the antagonist (lower). (C) Mean effects of DHPG on MLI firing. Facilitation was significantly blocked by CPCCOEt (*p<0.05, *n* = 5). (D) Puff-application of DHPG (arrowhead) induced slow inward currents under control conditions. The inward current was blocked by CPCCOEt (100 µM) and a competitive mGluR1 antagonist LY367385 (100 µM). Average traces obtained from three continuous current events are shown. (E) Time courses of the effects of the mGluR1 antagonists CPCCOEt (black circles, 100 µM, *n* = 5) and LY367385 (gray circles, 100 µM, *n* = 4). Black and gray bars indicate the time periods of CPCCOEt and LY367385 application, respectively.

To examine the effects of the group I mGluR agonist DHPG on MLIs, we recorded spontaneous action potentials from MLIs by loose cell-attached recordings. Under control conditions, the firing rate was variable among MLIs (5.2±1.2 Hz, ranging from 0 to 8 Hz, *n* = 6). Puff-application of DHPG (300 µM) transiently increased the firing rate to 19.3±2.9 Hz (*n* = 6). The increase of the firing rate was 312±47% of the control (excluding one silent cell in the control, *p*<0.05, n = 5, Fig. 1A and C). Perfusion of the non-competitive mGluR1 antagonist CPCCOEt (30 µM) significantly inhibited this DHPG-induced facilitation of firing frequency (141±28% of control, *n* = 6, Fig. 1B and C) as previously reported in rat cerebellar MLIs [14]. In cerebellar PCs, mGluR1 activation induces an excitatory slow inward current [18]–[20] through activation of a nonselective cation channel, the TRPC3 channel [21]–[25]. To address whether mGluR1 activation in MLIs triggers an excitatory inward current, we applied whole-cell voltage-clamp recordings to mouse cerebellar MLIs. Puff-application of DHPG to MLIs held at −70 to −65 mV induced an inward current (Fig. 1D) as reported in rat cerebellar MLIs [14]. The DHPG-induced inward current was reduced by CPCCOEt (100 µM) from 22.8±4.2 pA to 5.3±1.0 pA (24±4% of control, *p*<0.05, *n* = 5, Fig. 1D and E). In addition, a competitive mGluR1 antagonist LY367385 (100 µM) reduced the amplitude of this inward current from 21.6±3.5 pA to 2.4±0.5 pA (11±5% of control, *p*<0.001, *n* = 4, Fig. 1D and E), suggesting that the DHPG-induced inward current is elicited by mGluR1 activation in mouse cerebellar MLIs.

**Figure 2:**
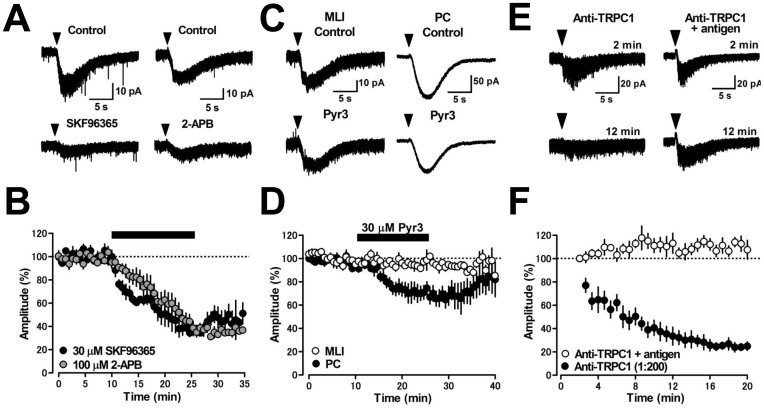
Induction of mGluR1-mediated inward current requires activation of TRPC1 but not TRPC3 in MLIs. (A) Nonselective TRPC channel blockers SKF96365 (30 µM) and 2-APB (100 µM) reduced the DHPG-induced inward current. (B) Time courses of the effects of SKF96365 (black circles, 30 µM, *n* = 4) and 2-APB (gray circles, 100 µM, *n* = 5) on the DHPG-induced inward current. A black bar indicates the time of drug application. (C) A TRPC3 blocker Pyr3 (30 µM) suppressed the DHPG-induced inward current in PCs (right) but not in MLIs (left). (D) Time courses of the effects of Pyr3 on the DHPG-induced inward current in MLIs (white circles, *n* = 5) and in PCs (black circles, *n* = 6). (E) Dialysis of an anti-TRPC1 antibody (1∶200 dilution) into MLIs through patch pipettes markedly reduced the DHPG-induced inward current (left), while dialysis of the antibody preincubated with its antigenic peptide did not (right). The current traces were obtained at the indicated time points (2 and 12 min) after establishing whole-cell configuration. (F) Time course of the effect of the anti-TRPC1 antibody on the DHPG-induced inward current (black circles, *n* = 6) compared to control experiments using the antibody preincubated with its antigenic peptide (white circles, n = 5).

The intracellular signaling mechanisms underlying this DHPG-induced inward current in MLIs has not been elucidated. Group I mGluRs are linked to store-operated channels formed by TRPC channel subunits in other types of neurons [17], [21]–[26]. To examine whether TRPC channel activation is necessary for induction of the DHPG-induced inward current in MLIs, we applied nonselective TRPC channel blockers SKF96365 or 2-APB to cerebellar slices. SKF96365 (30 µM) reduced the amplitude of DHPG-induced inward currents from 25.4±1.4 to 9.1±1.4 pA (36±6% of control, *p*<0.01, *n* = 4, Fig. 2A and B), while 2-APB (100 µM) suppressed the DHPG-current from 41.0±6.7 pA to 18.1±5.1 pA (42±7% of control, *p*<0.01, *n* = 5, Fig. 2A and B). This result is consistent with the reduction of DHPG-induced Ca^2+^ transients by 2-APB in rat cerebellar MLIs [27]. The inhibitory effects of these TRPC channel blockers were not reversible within the time frame of the voltage-clamp recordings.

TRPC1 and TRPC3 are expressed abundantly in the rodent cerebellum [22], [28]. To examine if TRPC1 and TRPC3 contribute to the mGluR1-mediated inward current in MLIs, we tested the effects of a TRPC3 channel inhibitor Pyr3 [29] or an anti-TRPC1 antibody [30] on the DHPG-induced inward current. Perfusion of Pyr3 (30 µM) did not alter the DHPG-evoked current amplitude in MLIs from 27.0±8.7 pA to 25.5±8.3 pA (95±1% of control, *p* = 0.059, *n* = 5, Fig. 2C and D), but did significantly suppress the DHPG-evoked current in PCs from 279±131 pA to 191±95 pA (63±6% of control, *p*<0.05, *n* = 6, Fig. 2C and D) [24], [25]. Dyalysis of an anti-TRPC1 antibody at 1∶200 dilution [30] into MLIs markedly decreased the current amplitude to 24±3% of the control (20.3±1.4 pA at 2 min vs. 5.0±0.8 pA at 20 min after whole-cell configuration, *p*<0.001, *n* = 6, Fig. 2E and F), while the inhibitory effect of the anti-TRPC1 antibody was blocked by preincubation with its control peptide antigen (23.2±4.7 pA at 2 min vs. 25.4±5.1 pA at 20 min after whole-cell configuration, *p* = 0.120, *n* = 5, Fig. 2E and F). These results suggest that TRPC channels consisting of TRPC1 isoforms contribute to the induction of mGluR1-mediated inward current in MLIs.

**Figure 3:**
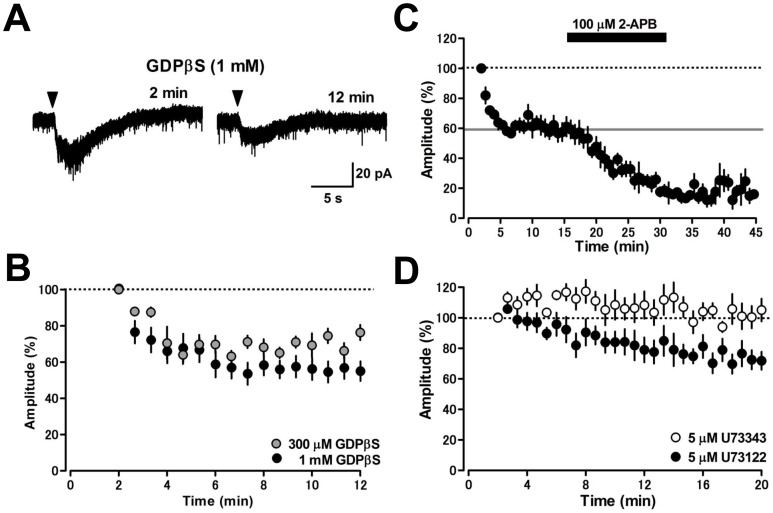
Activation of G proteins and PLC is required for induction of a component of the mGluR1-mediated inward current in MLIs. (A) Dialysis of a nonhydrolyzable G protein inactivator GDPβS (1 mM) into MLIs through patch pipettes decreased the DHPG-induced inward current gradually but did not inhibit the current completely. (B) Time courses of the effect of GDPβS on the DHPG-induced inward current. GDPβS reduced the current amplitude in a dose-dependent manner (gray circles, 300 µM, *n* = 5; black circles, 1 mM, *n* = 7). (C) The G protein-independent component of the DHPG-induced inward current was blocked by the nonselective TRPC channel blocker 2-APB (100 µM, *n* = 5). The DHPG-current was recorded by whole-cell recordings with patch pipettes containing 1 mM GDPβS. The gray line indicates the average percent value obtained during 12–16 min (59%). (D) A minor component of the mGluR1-mediated inward current was dependent on PLC activation. Infusion of a PLC inhibitor U73122 (black circles, 5 µM, *n* = 5) decreased the DHPG-induced inward current moderately, while U73343, an inactive analog of U73122, at the same concentration did not alter the current amplitude (white circles, *n* = 5).

Although induction of the mGluR1-mediated inward currents in cerebellar PCs is exclusively dependent on the activation of G proteins [18], [19], the inward currents are G protein-independent in neurons of the hippocampus and midbrain [15], [26]. To examine whether the DHPG-induced inward current in MLIs is G protein-dependent, we dialyzed the nonhydrolyzable G protein inactivator GDPβS into MLIs through patch pipettes. The agent dose-dependently decreased the amplitude of DHPG-induced inward current (Fig. 3B). GDPβS (1 mM) reduced the current amplitude from 27.6±2.7 pA at 2 min to 14.3±1.0 pA at 12 min after whole-cell configuration (54±5% of control, *p*<0.01, *n* = 7, Fig. 3A and B), while the current was not reduced over time by standard pipette solution without the agent (27.1±3.4 pA at 2 min vs. 28.1±3.7 pA at 12 min after whole-cell configuration, *p* = 0.148, *n* = 15). At the same concentration, GDPβS suppressed a larger fraction of mGluR1-mediated inward current in cerebellar PCs [18], [19], suggesting that the DHPG-induced inward current in MLIs is dependent on both G protein-dependent and -independent signaling pathways. Alternatively, it is possible that GDPβS (1 mM) does not completely inactivate the G protein-dependent pathway in MLIs. To clarify whether 1 mM GDPβS infused into MLIs completely blocks G proteins, we examined the effect of GDPβS on G_i/o_–dependent outward currents evoked by stimulation of GABA_B_ receptors [20], [31]. Bath-application of the GABA_B_ receptor agonist baclofen (3 µM) produced an outward current (6.8±0.7 pA, *n* = 5) that was completely blocked by infusion of GDPβS (1 mM) in the patch pipette (−0.6±1.2 pA at 10 min after whole-cell configuration, *p*<0.001, unpaired Student's *t*-test, *n* = 5), indicating that infusion of 1 mM GDPβS completely blocked G protein activation in MLIs. These results suggest that approximately half of the mGluR1-mediated inward current at peak amplitude is G protein-dependent. To examine whether the induction of G protein-independent mGluR1-mediated inward current requires the activation of TRPC channels, we bath-applied 2-APB 16 min after whole-cell configuration under conditions of G protein blockade (infusion of 1 mM GDPβS). 2-APB (100 µM) decreased the G protein-independent inward current from 23.7±5.4 pA to 7.3±1.9 pA (*p*<0.05, n = 5, Fig. 3C), suggesting that the mGluR1-mediated TRPC1-current indeed consists of both G protein-dependent and -independent components.

In cerebellar PCs, a major component of the mGluR1-mediated inward current is independent of the activation of PLC [18]–[21], but this current is absent in PCs of PLCβ4-deficient mice [32]. To test whether PLC activation is required for the induction of DHPG-induced inward current in MLIs, we infused the PLC inhibitor U73122 into MLIs through patch pipettes. U73122 (5 µM) decreased the DHPG-induced inward current from 28.4±7.1 pA at 2 min to 19.4±3.7 pA at 20 min after whole-cell configuration (72±6% of control, *p*<0.05, *n* = 5, Fig. 3D), while the same concentration of U73343, an inactive analog of U73122, did not affect the current amplitude (from 20.8±3.7 pA at 2 min to 20.5±3.0 pA at 20 min after whole-cell configuration, 102±7% of control, *p* = 0.867, *n* = 5, Fig. 3D). These data suggest that approximately 30% of the DHPG-induced inward current in MLIs requires PLC activation.

**Figure 4:**
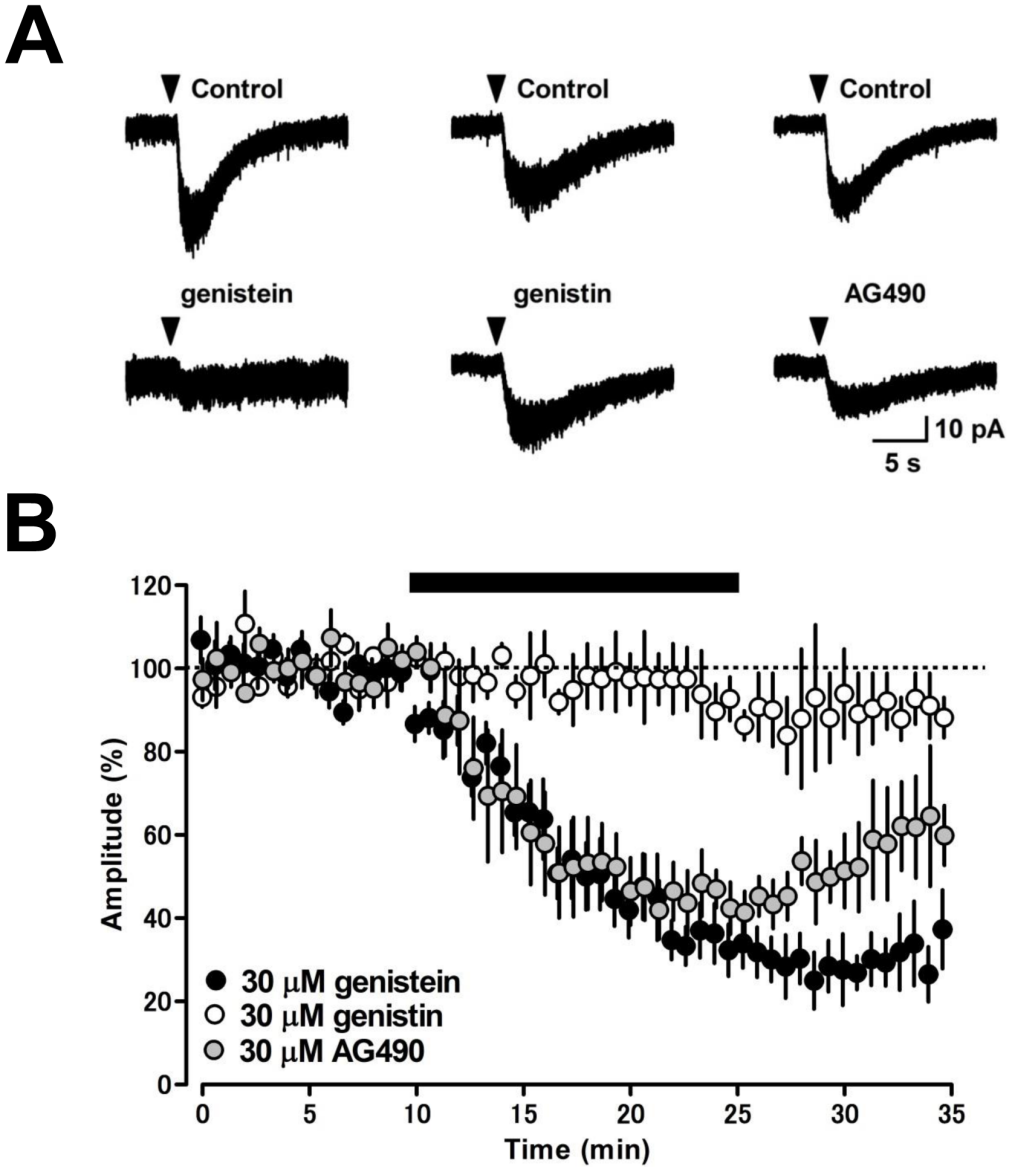
Effects of tyrosine kinase inhibitors on DHPG-induced inward current in MLIs. (A) Representative examples of the DHPG-induced inward current. Averaged traces were obtained from three continuous current events. Genistein (30 µM, left), a genistein-inactive analogue genistin (30 µM, center), and AG490 (30 µM, right) were bath-applied for 15 min. (B) Time courses of the effects of the tyrosine kinase inhibitors, genistein and AG490, and genistin on the DHPG-induced inward current. The DHPG-current was suppressed by genistein (black circles, *n* = 4) and AG490 (gray circles, *n* = 5), but not by genistin (white circles, *n* = 4). A black bar indicates the time period of drug application.

**Figure 5:**
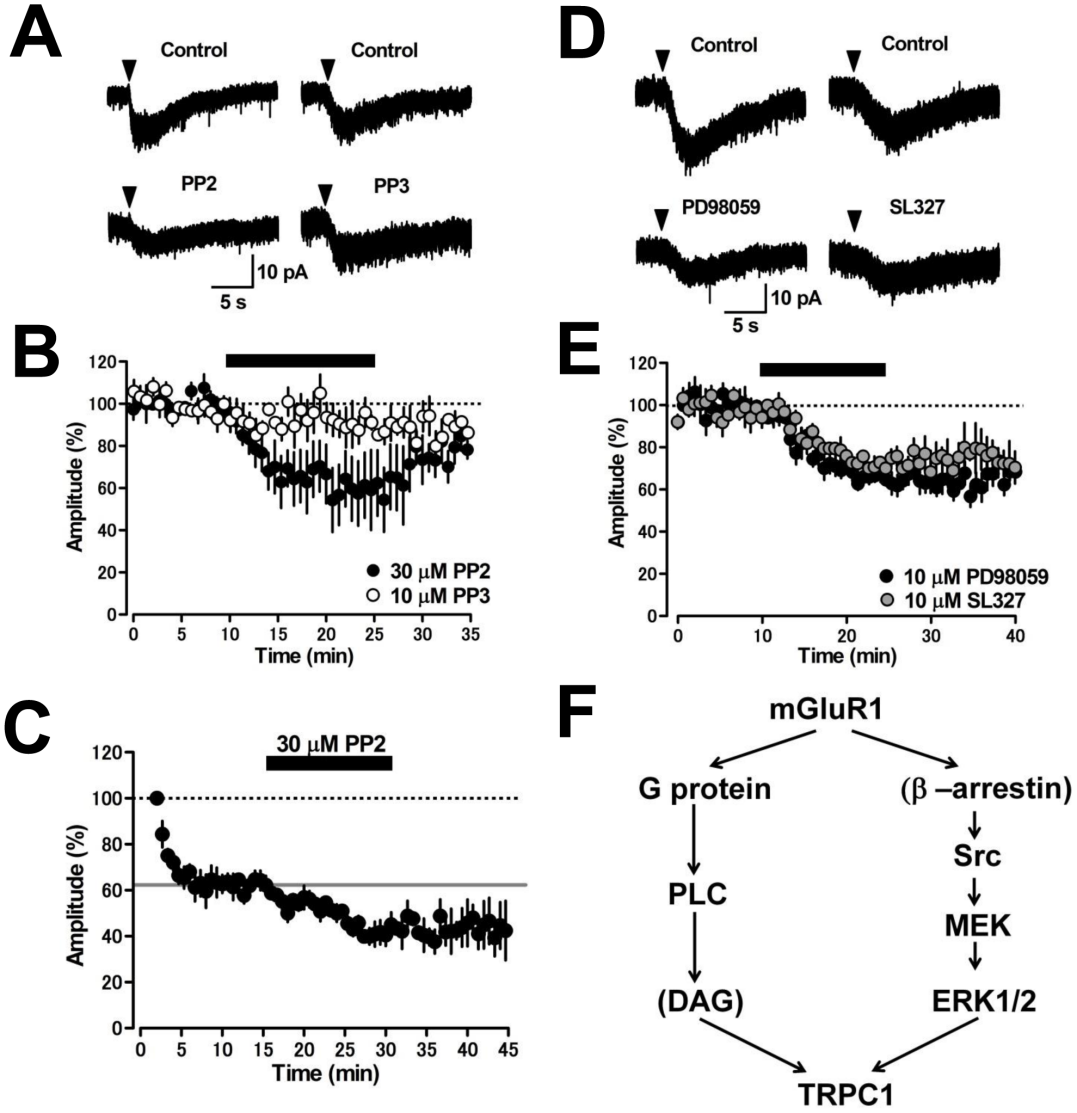
Effects of Src tyrosine kinase inhibitor and MEK inhibitors on the DHPG-induced inward current in MLIs. (A) Representative traces under control conditions and in the presence of the Src tyrosine kinase inhibitor PP2 (30 µM) and the inactive PP2 analog PP3 (10 µM) are shown. Average traces were obtained from three continuous current events. (B) Time courses of the effects of PP2 and PP3 on the DHPG-induced inward current. The DHPG-current was suppressed by PP2 (black circles, *n* = 5) but not by PP3 (white circles, *n* = 4). A black bar indicates the time period of drug application. (C) The G protein-independent component of the DHPG-induced inward current was blocked by the Src tyrosine kinase inhibitor PP2 (30 µM). The DHPG-induced currents were recorded by whole-cell recordings with patch pipettes containing 1 mM GDPβS. The gray line indicates the average percent value during 12–16 min (63%). (D) MEK inhibitors PD98059 (10 µM) and SL327 (10 µM) reduced the DHPG-induced inward current. Averaged traces obtained from three continuous current events are shown. (E) Time courses of the effects of PD98059 (black circles, 10 µM, *n* = 5) and SL327 (gray circles, 10 µM, *n* = 4) on the DHPG-induced inward current (*n* = 5). A black bar indicates the time point for application of the drugs. (F) Schematic diagram of signaling pathways required for the induction of mGluR1-mediated inward current in MLIs. The activation of mGluR1 opens TRPC1 channels through both G protein-dependent and G-protein-independent Src–ERK1/2 signaling pathways. DAG, diacylglycerol.

In hippocampal neurons, activation of Src PTK family is required for the induction of mGluR1-mediated inward current [15], [17]. To assess the contribution of Src signaling in MLIs, we first applied the broad-spectrum PTK blocker genistein to cerebellar slices for 15 min. Genistein (30 µM) markedly decreased the amplitude of the DHPG-induced inward current from 23.3±4.9 pA to 4.7±0.3 pA (20±1% of control, *p*<0.05, *n* = 4, Fig. 4A and B), while the same concentration of genistin, an inactive form of genistein, did not alter the current amplitude (from 23.4±2.8 pA to 22.0±2.2 pA, 94±10% of control, *p* = 0.171, *n* = 4, Fig. 4A and B). The other selective PTK inhibitor AG490 (30 µM) also reduced the amplitude of the DHPG-induced inward current from 23.4±4.7 pA to 9.1±2.8 pA (39±12% of control, *p*<0.01, *n* = 5, Fig. 4A and B). Furthermore, we examined the contribution of Src family kinases using the selective Src inhibitor PP2. Bath application of PP2 at 10 µM decreased the amplitude of DHPG-induced inward currents to 65±11% of the control response (*p*<0.01, *n* = 5), and PP2 at 30 µM reduced the inward current from 25.2±3.4 pA to 14.0±2.7 pA (53±6% of control, *p*<0.001, *n* = 5, Fig. 5A and B). PP3, an inactive analog of PP2, did not significantly alter the inward current at 10 µM from 16.7±2.1 pA to 16.2±1.4 pA (97±9% of control, *p* = 0.562, *n* = 4, Fig. 5A and B). To examine whether the G protein-independent DHPG-induced inward current requires activation of Src, we bath-applied PP2 following G protein blockade by intracellular infusion of GDPβS (1 mM). PP2 (30 µM) significantly reduced the G protein-independent inward current component from 15.2±2.6 pA to 9.6±2.5 pA (*p*<0.01, n = 5, Fig. 5C), suggesting that the G protein-independent mGluR1-mediated inward current is induced by Src activation. To examine whether activation of ERK1/2, which is downstream of the Src family kinase, is involved in the induction of the mGluR1-mediated inward current, we applied mitogen extracellular regulating kinase (MEK) inhibitors PD98059 and SL327 to MLIs [17], [33]. PD98059 (10 µM) gradually reduced the amplitude of DHPG-induced inward current from 21.4±4.7 pA to 11.9±3.4 pA (56±16% of control, *p*<0.01, *n* = 5, Fig. 5D and E) 15 min after application. In addition, SL327 (10 µM) gradually reduced the current amplitude from 24.6±2.5 pA to 16.3±1.9 pA (66±1% of control, *p*<0.01, *n* = 4, Fig. 5D and E) 15 min after application. These results suggest that the Src-ERK1/2 signaling pathway plays a crucial role in the induction of mGluR1-mediated inward current in MLIs.

**Figure 6:**
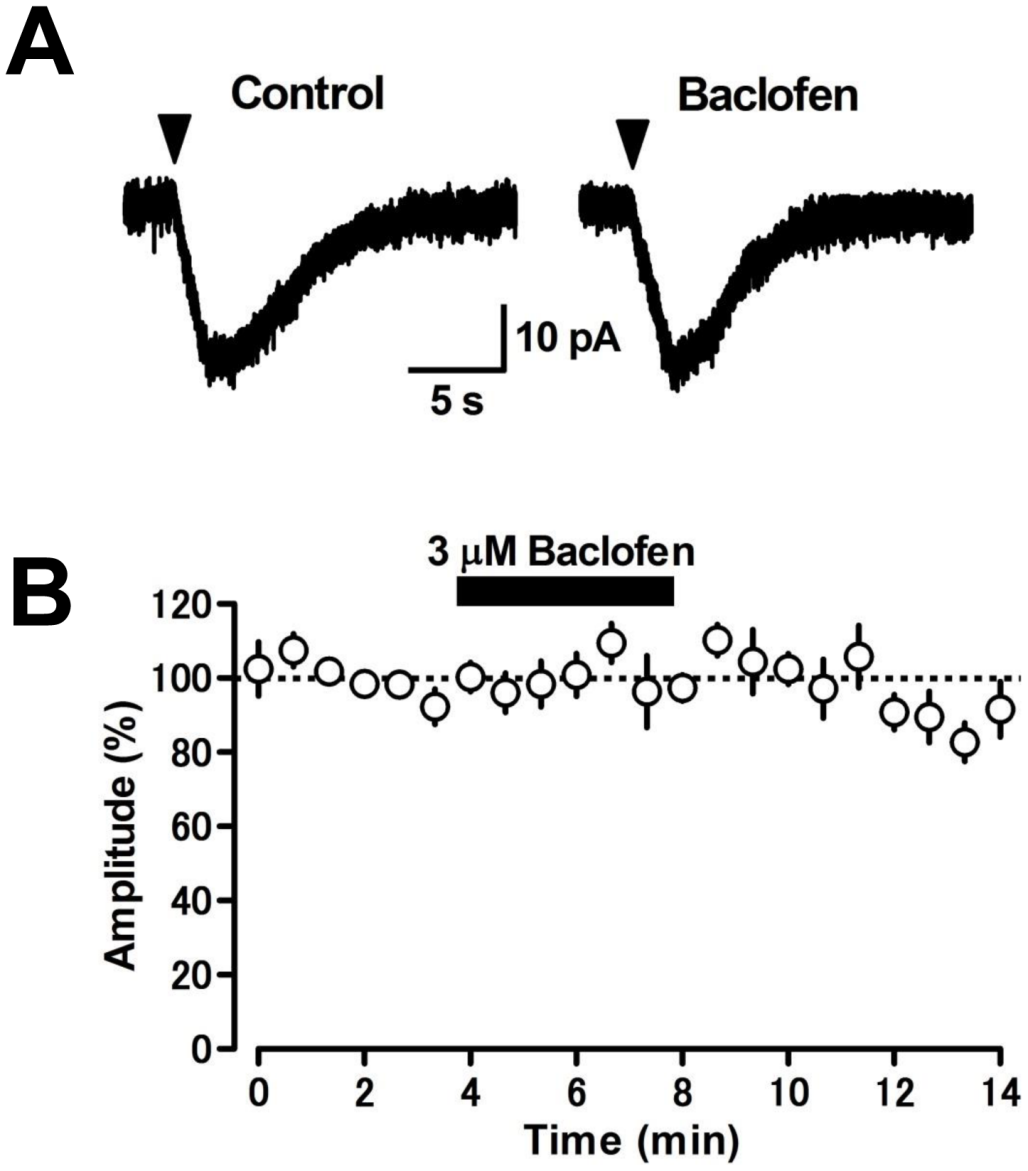
GABA_B_ receptor activation has no effect on the DHPG-induced inward current in MLIs. (A) Application of the GABA_B_ receptor agonist baclofen (3 µM) did not alter the DHPG-induced inward current. Averaged traces obtained from three continuous current events are shown. (B) Time course of the effect of baclofen on the DHPG-induced inward current (*n* = 6). The amplitude of the DHPG-evoked current is expressed as a percentage of the averaged control over the 4-min period before baclofen application.

Our previous study demonstrated that GABA_B_ receptor activation enhances the mGluR1-mediated excitatory inward current in cerebellar PCs [20]. Moreover, such cross-talk is suggested in MLIs because the mGluR1-induced reduction in the surface expression of GluR2-contaning (Ca^2+^-permeable) AMPA receptors was enhanced by GABA_B_ receptor stimulation in rat cerebellar stellate cells [34]. We examined whether GABA_B_ receptor activation enhances the mGluR1-mediated inward current in MLIs. Bath-application of the GABA_B_ receptor agonist baclofen (3 µM) for 4 min did not significantly change the amplitude of DHPG-induced inward current (from 18.9±2.3 pA to 19.1±2.4 pA, 101±13% of control, *p* = 0.729, *n* = 6, Fig. 6A and B). Thus, no cross-talk was detected between downstream signaling activated by mGluR1 and GABA_B_ receptors in MLIs.

## Discussion

We describe the intracellular signaling mechanisms underlying the mGluR1-mediated inward current in mouse cerebellar MLIs. The mGluR1-mediated inward current is mediated by TRPC channels, including the TRPC1 isoform, which are activated by the two signaling pathways shown in Figure 5F, one G protein-dependent and the other G protein-independent and Src–ERK1/2-dependent. By contrast, the latter signaling pathway is not necessary for the mGluR1-mediated inward current in cerebellar PCs [18]–[21]. Furthermore, GABA_B_ receptor activation has no effect on the mGluR1-mediated inward current in MLIs, suggesting that there is no cross-talk between mGluR1 and GABA_B_ receptors in MLIs as observed in PCs [20].

Over the past decade, slow excitatory responses induced by activation of group I mGluRs have been examined in neurons of several brain areas. Some of the responses evoked by group I mGluRs are G protein-dependent [18], [19], [21] and others G protein-independent [15]–[17]. In cerebellar PCs, this mGluR1-mediated inward current is entirely G protein-dependent but not dependent on PLC activation [18]–[21]. Moreover, the selective Src inhibitor PP1 did not suppress the inward current (it increased DHPG-induced excitatory electrical responses) [35], [36], suggesting that activation of Src family kinase is not necessary for induction of mGluR1-mediated inward current in PCs.

By contrast, we found that only approximately half of the mGluR1-mediated inward current was induced through G protein-dependent pathway in MLIs. Our previous studies have shown that the mGluR1-mediated inward current in MLIs is PLC-independent, as the selective PLC inhibitor U73122 did not alter DHPG-induced facilitation of spontaneous IPSCs in PCs [37]. Thus, only a small fraction of the DHPG-induced inward current in MLIs requires PLC activation. This result is consistent with a previous report showing that U73122 does not suppress the first Ca^2+^ transient evoked by the mGluR1-mediated activation of TRPC channels in rat cerebellar MLIs [27]. Taken together, a majority of the mGluR1-mediated inward current in MLIs is PLC-independent, similar to cerebellar PCs [18]–[21] and hippocampal CA1 pyramidal neurons [38]. In the presence of the G protein inhibitor in MLIs, 2-APB did not suppress the DHPG-induced inward current completely, implying that the G protein-independent and TRPC-independent component may be involved in the inward current. It has been reported that the activation of mGluR1 opens ionotropic glutamate receptors [25], [39], [40]. However, we can rule out this possibility because the nonselective ionotropic glutamate receptor antagonist kynurenic acid was added to ACSF throughout the experiments. Therefore, transporters or ion channels other than TRPC channels and ionotropic glutamate receptors may be activated by the mGluR1-mediated and G protein-independent pathways. An alternative reason for the partial and irreversible effect of the TRPC channel inhibitors may be the location of MLIs that we recorded, i.e., they were not located on the surface of cerebellar slices but at the depth of approximately 100 µm. Therefore, the drugs have a tendency to take a longer time to reach the recorded cells, and they also require a longer time to be washed out. Indeed, in other slice experiments, their inhibitory effects of the drugs are either reversed very slowly or not reversible within the time frame of the voltage-clamp recordings [41], [42]. Under G protein-inhibition, the selective Src inhibitor PP2 suppressed the amplitude of the DHPG-induced inward current, suggesting that Src activation, possibly mediated by β-arrestins [40], [43], [44], contributes to a component of the mGluR1-mediated inward current in MLIs.

We observed marked inhibition of mGluR1-mediated inward current in the presence of the broad-spectrum PTK blocker genistein; this is consistent with a previous study showing that tyrosine phosphorylation regulates activation of the G protein G_q/11_ [45]. In hippocampal CA3 pyramidal neurons, mGluR1-mediated excitatory responses are independent of G protein activation but dependent on Src activation [15]. Furthermore, increasing intracellular Ca^2+^ rescues the coupling between group I mGluRs and the TRP-like conductance under conditions of G protein inactivation [46]. In addition, we demonstrated that activation of ERK1/2 is required for the induction of at least part of the mGluR1-mediated inward current in MLIs. In rat hippocampal oriens/alveus interneurons, ERK1/2 activation is involved in the mGluR1/5-mediated Ca^2+^ signal and the inward current [17], [47]. On the other hand, the mGluR1-mediated slow EPSCs in mouse cerebellar PCs do not require activation of the MEK−ERK1/2 pathway [48], another difference in the mechanism of mGluR1-induced signal transduction between MLIs and PCs.

Stimulation of mGluR1 in PCs induces an excitatory slow inward current through activation of nonselective cation channels including TRPC3 [21]–[25]. Immunohistochemical studies indicate that both PCs and MLIs express TRPC3 cation channels [22], and MLIs appear to express TRPC6 more abundantly than TRPC3 [28]. TRPC3 and TRPC6 belong to the same structural and functional subfamily of TRPCs [49]; therefore, it is likely that MLIs express heteromeric tetramers, including both TRPC3 and TRPC6. However, the TRPC3 inhibitor Pyr3 did not alter the DHPG-induced inward current in MLIs, while intracellular infusion of anti-TRPC1 antibody markedly reduced the inward current. Thus, MLIs express TRPC channels mainly composed of the TRPC1 isoform.

It has been reported that the activation of either mGluR1 or GABA_B_ receptor can reduce the number of Ca^2+^-permeable AMPA receptors in MLIs by increasing intracellular Ca^2+^ [34]. Simultaneous activation of both mGluR1 and GABA_B_ receptors is assumed to additively or synergistically affect the reduction in Ca^2+^-permeable AMPA receptors. In PCs, the simultaneous activation enhances the mGluR1-mediated inward current [20]. However, in the present study, we detected no change in the amplitude of the mGluR1-mediated inward current in MLIs during GABA_B_ receptor activation (as would be expected if there were in fact cross-talk between mGluR1 and GABA_B_ receptor signaling). One reason for the lack of cross-talk may be the distinct subcellular distributions of these receptors in MLIs. Alternatively, the signaling pathways activated in downstream of mGluR1 may differ between MLIs and PCs.

MLIs receive excitatory synaptic inputs from parallel fibers, and glutamate released by high-frequency stimulation of parallel fibers activates both ionotropic glutamate receptors and the group I mGluRs [50], which subsequently facilitate spontaneous firing in MLIs [14], [27]. At MLI postsynaptic sites, activation of group I mGluRs and Ca^2+^-permeable AMPA receptors drives an AMPA receptor subunit switch that reduces surface expression of Ca^2+^-permeable AMPA receptors [34]. On the other hand, even low-frequency stimulation of parallel fibers can activate group I mGluRs and induce long-term synaptic depression at parallel fiber–stellate cell synapses [51]. ERK1/2 activated by the mGluR1-mediated signal transduction pathway as observed here may contribute to synaptic plasticity at parallel fiber–MLI synapses because in general ERK1/2 activation at postsynaptic sites contributes to the induction of synaptic plasticity [52]. At parallel fiber–PC synapses, activation of postsynaptic ERK1/2 mediates long-term synaptic depression [48], [53], [54]. MLIs regulate the firing patterns of PCs by feedforward inhibition [4]–[7], [9], [10], and are essential regulators of cerebellar signal processing for motor learning [3], [8], [9]. Thus, mGluR1-mediated facilitation of firing in MLIs not only regulates PC activity but also elicits long-term plasticity, which is associated with the magnitude of the feedforward inhibition.

## References

[1] Bartos M, Vida I, Jonas P (2007) Synaptic mechanisms of synchronized gamma oscillations in inhibitory interneuron networks. Nat Rev Neurosci 8: 45–56. 17180162 10.1038/nrn2044

[2] Kullmann DM, Lamsa KP (2007) Long-term synaptic plasticity in hippocampal interneurons. Nat Rev Neurosci 8: 687–699. 17704811 10.1038/nrn2207

[3] De Zeeuw CI, Hoebeek FE, Bosman LW, Schonewille M, Witter L, et al (2011) Spatiotemporal firing patterns in the cerebellum. Nat Rev Neurosci 12: 327–344. 21544091 10.1038/nrn3011

[4] Häusser M, Clark BA (1997) Tonic synaptic inhibition modulates neuronal output pattern and spatiotemporal synaptic integration. Neuron 19: 665–678. 9331356 10.1016/s0896-6273(00)80379-7

[5] Mittmann W, Koch U, Häusser M (2005) Feed-forward inhibition shapes the spike output of cerebellar Purkinje cells. J Physiol 563: 369–378. 15613376 10.1113/jphysiol.2004.075028 PMC1665592

[6] Smith SL, Otis TS (2005) Pattern-dependent, simultaneous plasticity differentially transforms the input-output relationship of a feedforward circuit. Proc Natl Acad Sci U S A 102: 14901–14906. 16199519 10.1073/pnas.0505028102 PMC1253560

[7] Barmack NH, Yakhnitsa V (2008) Functions of interneurons in mouse cerebellum. J Neurosci 28: 1140–1152. 18234892 10.1523/JNEUROSCI.3942-07.2008 PMC6671404

[8] Obata K, Hirono M, Kume N, Kawaguchi Y, Itohara S, et al (2008) GABA and synaptic inhibition of mouse cerebellum lacking glutamate decarboxylase 67. Biochem Biophys Res Commum 370: 429–433. 10.1016/j.bbrc.2008.03.110 18384748

[9] Wulff P, Schonewille M, Renzi M, Viltono L, Sassoé-Pognetto M, et al (2009) Synaptic inhibition of Purkinje cells mediates consolidation of vestibule-cerebellar motor learning. Nat Neurosci 12: 1042–1049. 19578381 10.1038/nn.2348 PMC2718327

[10] Dizon MJ, Khodakhah K (2011) The role of interneurons in shaping Purkinje cell responses in the cerebellar cortex. J Neurosci 31: 10463–10473. 21775592 10.1523/JNEUROSCI.1350-11.2011 PMC3314287

[11] Hartmann J, Konnerth A (2009) Mechanisms of metabotropic glutamate receptor-mediated synaptic signaling in cerebellar Purkinje cells. Acta Physiol 195: 79–90.

[12] Luscher C, Huber KM (2010) Group I mGluR-dependent synaptic long-term depression: mechanisms and implications for circuitry and disease. Neuron 65: 445–459. 20188650 10.1016/j.neuron.2010.01.016 PMC2841961

[13] Llano I, Marty A (1995) Presynaptic metabotropic glutamatergic regulation of inhibitory synapses in rat cerebellar slices. J Physiol 486: 163–176. 7562633 10.1113/jphysiol.1995.sp020800 PMC1156506

[14] Karakossian MH, Otis TS (2004) Excitation of cerebellar interneurons by group I metabotropic glutamate receptors. J Neurophysiol 92: 1558–1565. 15152021 10.1152/jn.00300.2004 PMC2676872

[15] Heuss C, Scanziani M, Gähwiler BH, Gerber U (1999) G-protein-independent signaling mediated by metabotropic glutamate receptors. Nat Neurosci 2: 1070–1077. 10570483 10.1038/15996

[16] Gee CE, Lacaille JC (2004) Group I metabotropic glutamate receptor actions in oriens/alveus interneurons of rat hippocampal CA1 region. Brain Res 1000: 92–101. 15053957 10.1016/j.brainres.2003.11.046

[17] Topolnik L, Azzi M, Morin F, Kougioumoutzakis A, Lacaille JC (2006) mGluR1/5 subtype-specific calcium signaling and induction of long-term potentiation in rat hippocampal oriens/alveus interneurones. J Physiol 575: 115–131. 16740609 10.1113/jphysiol.2006.112896 PMC1819425

[18] Hirono M, Konishi S, Yoshioka T (1998) Phospholipase C-independent group I metabotropic glutamate receptor-mediated inward current in mouse Purkinje cells. Biochem Biophys Res Commum 251: 753–758. 10.1006/bbrc.1998.9465 9790982

[19] Tempia F, Miniaci MC, Anchisi D, Strata P (1998) Postsynaptic current mediated by metabotropic glutamate receptors in cerebellar Purkinje cells. J Neurophysiol 80: 520–528. 9705447 10.1152/jn.1998.80.2.520

[20] Hirono M, Yoshioka T, Konishi S (2001) GABA_B_ receptor activation enhances mGluR-mediated responses at cerebellar excitatory synapses. Nat Neurosci 4: 1207–1216. 10.1038/nn764 11704764

[21] Glitsch MD (2010) Activation of native TRPC3 cation channels by phospholipase D. FASEB J. 24: 318–325. 10.1096/fj.09-134973 19741172

[22] Hartmann J, Dragicevic E, Adelsberger H, Henning HA, Sumser M, et al (2008) TRPC3 channels are required for synaptic transmission and motor coordination. Neuron 59: 392–398. 18701065 10.1016/j.neuron.2008.06.009 PMC2643468

[23] Becker EB, Oliver PL, Glitsch MD, Banks GT, Achilli F, et al (2009) A point mutation in TRPC3 causes abnormal Purkinje cell development and cerebellar ataxia in moonwalker mice. Proc Natl Acad Sci U S A 106: 6706–6711. 19351902 10.1073/pnas.0810599106 PMC2666615

[24] Nelson C, Glitsch MD (2012) Lack of kinase regulation of canonical transient receptor potential 3 (TRPC3) channel-dependent currents in cerebellar Purkinje cells. J Biol Chem 287: 6326–6335. 22207762 10.1074/jbc.M111.246553 PMC3307326

[25] Ady V, Perroy J, Tricoire L, Piochon C, Dadak S, et al (2014) Type 1 metabotropic glutamate receptors (mGlu1) trigger the gating of GluD2 delta glutamate receptors. EMBO reports 15: 103–109. 24357660 10.1002/embr.201337371 PMC4303454

[26] Tozzi A, Guatteo E, Caputi L, Bernardi G. Mercuri NB (2001) Group I mGluRs coupled to G proteins are regulated by tyrosine kinase in dopamine neurons of the rat midbrain. J Neurophysiol 85: 2490–2497. 11387395 10.1152/jn.2001.85.6.2490

[27] Collin T, Franconville R, Ehrlich BE, Llano I (2009) Activation of metabotropic glutamate receptors induces periodic burst firing and concomitant cytosolic Ca^2+^ oscillations in cerebellar interneurons. J Neurosci 29: 9281–9291. 19625518 10.1523/JNEUROSCI.1865-09.2009 PMC6665558

[28] Huang WC, Young JS, Glitsch MD (2007) Changes in TRPC channel expression during postnatal development of cerebellar neurons. Cell Calcium 42: 1–10. 17141310 10.1016/j.ceca.2006.11.002

[29] Kiyonaka S, Kato K, Nishida M, Mio K, Numaga T, et al (2009) Selective and direct inhibition of TRPC3 channels underlies biological activities of a pyrazole compound. Proc Natl Acad Sci U S A 106: 5400–5405. 19289841 10.1073/pnas.0808793106 PMC2664023

[30] Saleh SN, Albert AP, Peppiatt-Wildman CM, Large WA (2008) Diverse properties of store-operated TRPC channels activated by protein kinase C in vascular myocytes. J Physiol 586: 2463–2476. 18356201 10.1113/jphysiol.2008.152157 PMC2408673

[31] Bichet D, Haass FA, Jan LY (2003) Merging functional studies with structures of inward-rectifier K^+^ channels. Nat Rev Neurosci 4: 957–967. 14618155 10.1038/nrn1244

[32] Sugiyama T, Hirono M, Suzuki K, Nakamura Y, Aiba A, et al (1999) Localization of phospholipase Cβ isozymes in the mouse cerebellum. Biochem Biophys Res Commum 265: 473–478. 10.1006/bbrc.1999.1628 10558892

[33] Atkins CM, Selcher JC, Petraitis JJ, Trzaskos JM, Sweatt JD (1998) The MAPK cascade is required for mammalian associative learning. Nat Neurosci 1: 602–609. 10196568 10.1038/2836

[34] Kelly L, Farrant M, Cull-Candy SG (2009) Synaptic mGluR activation drives plasticity of calcium-permeable AMPA receptors. Nat Neurosci 12: 593–601. 19377472 10.1038/nn.2309

[35] Canepari M, Ogden D (2003) Evidence for protein tyrosine phosphatase, tyrosin kinase, and G-protein regulation of the parallel fiber metabotropic slow EPSC of rat cerebellar Purkinje neurons. J Neurosci 23: 4066–4071. 12764093 10.1523/JNEUROSCI.23-10-04066.2003 PMC6741078

[36] Auger C, Ogden D (2010) AMPA receptor activation controls type I metabotropic glutamate receptor signaling via a tyrosine kinase at parallel fiber-Purkinje cell synapses. J Physiol 588: 3063–3074. 20603338 10.1113/jphysiol.2010.191080 PMC2956945

[37] Kubota H, Hirono M, Obata K (2007) Tyrosine kinase is involved in the mGluR1-mediated inward current in the cerebellar molecular layer interneurons. Neurosci Res 58: S196.

[38] Ireland DR, Abraham WC (2002) Group I mGluRs increase excitability of hippocampal CA1 pyramidal neurons by a PLC-independent mechanism. J Neurophysiol 88: 107–116. 12091536 10.1152/jn.2002.88.1.107

[39] Benquet P, Gee CE, Gerber U (2002) Two distinct signaling pathways upregulate NMDA receptor responses via two distinct metabotropic glutamate receptor subtypes. J Neurosci 22: 9679–9686. 12427823 10.1523/JNEUROSCI.22-22-09679.2002 PMC6757830

[40] Gerber U, Gee CE, Benquet P (2007) Metabotropic glutamate receptors: intracellular signaling pathways. Curr Opin Pharmacol 7: 56–61. 17055336 10.1016/j.coph.2006.08.008

[41] Tozzi A, Bengtson CP, Longone P, Carignani C, Fusco FR, et al (2003) Involvement of transient receptor potential-like channels in responses to mGluR-I activation in midbrain dopamine neurons. Eur J Neurosci 18: 2133–2145. 14622174 10.1046/j.1460-9568.2003.02936.x

[42] Sansone A, Hassenklöver T, Syed AS, Korsching SI, Manzini I (2014) Phospholipase C and diacylglycerol mediated olfactory responses to amino acids in the main olfactory epithelium of an amphibian. PloS ONE 9: e87721. 24489954 10.1371/journal.pone.0087721 PMC3905040

[43] Luttrell LM, Ferguson SSG, Daaka Y, Miller WE, Maudsley S, et al (1999) β-Arrestin-dependent formation of β_2_ adrenergic receptor-Src protein kinase complexes. Science 283: 655–661. 9924018 10.1126/science.283.5402.655

[44] Lefkowitz RJ, Shenoy SK (2005) Transduction of receptor signals by β-arrestins. Science 308: 512–517. 15845844 10.1126/science.1109237

[45] Umemori H, Inoue T, Kume S, Sekiyama N, Nagao M, et al (1997) Activation of the G protein Gq/11 through tyrosine phosphorylation of the α subunit. Science 276: 1878–1881. 9188537 10.1126/science.276.5320.1878

[46] Gee CE, Benquet P, Gerber U (2003) Group I metabotropic glutamate receptors activate a calcium-sensitive transient receptor potential-like conductance in rat hippocampus. J Physiol 546: 655–664. 12562994 10.1113/jphysiol.2002.032961 PMC2342598

[47] Camiré O, Lacaille JC, Topolnik L (2012) Dendritic signaling in inhibitory interneurons: local tuning via group I metabotropic glutamate receptors. Front Physiol 3: 259. 22934015 10.3389/fphys.2012.00259 PMC3429035

[48] Ito-Ishida A, Kakegawa W, Yuzaki M (2006) ERK1/2 but not p38 MAP kinase is essential for the long-term depression in mouse cerebellar slices. Eur J Neurosci 24: 1617–1622. 17004925 10.1111/j.1460-9568.2006.05055.x

[49] Dietrich A, Kalwa H, Rost BR, Gudermann T (2005) The diacylgylcerol-sensitive TRPC3/6/7 subfamily of cation channels: functional characterization and physiological relevance. Eur J Physiol 451: 72–80. 10.1007/s00424-005-1460-0 15971081

[50] Ito M (2006) Cerebellar circuitry as a neuronal machine. Prog Neurobiol 78: 272–303. 16759785 10.1016/j.pneurobio.2006.02.006

[51] Rancillac A, Crepel F (2004) Synapses between parallel fibers and stellate cells express long-term changes in rat cerebellum. J Physiol 554: 707–720. 14617674 10.1113/jphysiol.2003.055871 PMC1664787

[52] Sweatt JD (2004) Mitogen-activated protein kinases in synaptic plasticity and memory. Curr Opin Neurobiol 14: 311–317. 15194111 10.1016/j.conb.2004.04.001

[53] Kawasaki H, Fujii H, Gotoh Y, Morooka T, Shimohama S, et al (1999) Requirement for mitogen-activated protein kinase in cerebellar long term depression. J Biol Chem 274: 13498–13502. 10224117 10.1074/jbc.274.19.13498

[54] Endo S, Launey T (2003) ERKs regulate PKC-dependent synaptic depression and declustering of glutamate receptors in cerebellar Purkinje cells. Neuropharmacol 45: 863–872. 10.1016/s0028-3908(03)00210-7 14529724

